# Impact of Extracellular Matrix Components to Renal Cell Carcinoma Behavior

**DOI:** 10.3389/fonc.2020.00625

**Published:** 2020-04-28

**Authors:** Sandra Majo, Sarah Courtois, Wilfried Souleyreau, Andreas Bikfalvi, Patrick Auguste

**Affiliations:** ^1^Université de Bordeaux, Bordeaux, France; ^2^INSERM, U1035, Bordeaux, France; ^3^IIS Aragon, Hospital Universitario Miguel Servet, Zaragoza, Spain; ^4^INSERM, U1029, Pessac, France

**Keywords:** renal cell carcinoma, extracellular matrix, migration, invasion, metalloproteinases

## Abstract

Renal cell carcinoma (RCC) represents the main renal tumors and are highly metastatic. They are heterogeneous tumors and are subdivided in 12 different subtypes where clear cell RCC (ccRCC) represents the main subtype. Tumor extracellular matrix (ECM) is composed, in RCC, mainly of different fibrillar collagens, fibronectin, and components of the basement membrane such as laminin, collagen IV, and heparan sulfate proteoglycan. Little is known about the role of these ECM components on RCC cell behavior. Analysis from The Human Protein Atlas dataset shows that high collagen 1 or 4A2, fibronectin, entactin, or syndecan 3 expression is associated with poor prognosis whereas high collagen 4A3, syndecan 4, or glypican 4 expression is associated with increased patient survival. We then analyzed the impact of collagen 1, fibronectin 1 or Matrigel on three different RCC cell lines (Renca, 786-O and Caki-2) *in vitro*. We found that all the different matrices have little effect on RCC cell proliferation. The three cell lines adhere differently on the three matrices, suggesting the involvement of a different set of integrins. Among the 3 matrices tested, collagen 1 is the only component able to increase migration in the three cell lines as well as MMP-2 and 9 activity. Moreover, collagen 1 induces MMP-2 mRNA expression and is implicated in the epithelial to mesenchymal transition of two RCC cell lines via Zeb2 (Renca) or Snail 2 (Caki-2) mRNA expression. Taken together, our results show that collagen 1 is the main component of the ECM that enhances tumor cell invasion in RCC, which is important for the metastasic process.

## Introduction

Renal cell carcinoma (RCC) represents about 2% of all adult malignancies and 90% of all kidney tumors (1, 2). It is the most lethal urological tumor with ~40% of patient's dead due to disease progression (3). Most of RCCs are sporadic and only 4–5% are inherited. Moreover, RCCs are highly metastatic and 25–30% of patients have metastasis at the time of diagnosis. According to the 2004 WHO classification, 12 histological subtypes are recognized with 3 main represented by clear cell renal cell carcinoma, papillary renal cell carcinoma and chromophobe renal cell carcinoma (4).

Clear cell RCC (ccRCC), the most frequent subtype with a 75% incidence, originates from proximal tubule epithelium. Cells are characterized by a clear or, occasionally eosinophil granular cytoplasm (3, 4). In the majority of the ccRCC, the Von Hippel-Lindau (VHL) gene is inactivated. This inactivation includes gene mutation, promoter hypermethylation, loss of heterozygosity by allele deletion and concomitant alteration of the second gene. Loss of VHL deregulates and constitutively activates hypoxia-inducible factor HIF1α and HIF2α. The two transcription factors play a role in ccRCC, but seem to have opposite effects, HIF1α is acting as a tumor suppressor with an expression lost in 30–40% of tumors whereas HIF2α is acting as an oncoprotein. HIF1α and HIF2α are both implicated in angiogenesis, cell proliferation, metastasis, resistance against endoplamic reticulum (ER) and oxydative stresses (5). Nevertheless, a recent meta-analysis study from Kim and colleagues found no correlation between VHL inactivation and patient survival in ccRCC (6).

Papillary renal cell carcinoma (pRCC) is a less aggressive tumor, accounting for 10% of all RCC. They derived from distal tubule epithelium (3) and are organized in papillae with small cells arranged in a single layer (type 1 or basophilic) or with cells of higher nuclear grade, eosinophilic cytoplasm and pseudostratified nuclei (type 2 or eosinophilic) (4). Type 2 is considered as more aggressive than type 1 (7).

Chromophobe renal cell carcinoma is derived from intercalated cells of the collecting duct and represents 5% of all RCC. The tumor is composed of large cells with a clear reticulated cytoplasm and perinuclear halos, but in some variants the cytoplasm is eosinophilic. It is the less aggressive RCC subtype unless a sarcomatoid transformation occurs (3, 4).

The other RCC subtypes, such as the medullary subtype, represent <5% of all RCC and are rare.

In 1982, Fuhrman and al proposed a RCC grading system based on nuclear size and shape and on nucleolar prominence. The Fuhrman tumor grade (I–IV) is directly correlated to patient survival (8) and to metastasis (9). Nevertheless, in several studies on chromophobe RCC, no correlation between the Fuhrman nuclear grade and patient survival was found (10). Beyond the Fuhrman grade, some RCCs with extreme dedifferentiation called sarcomatoid RCC, undergo epithelial to mesenchymal transformation (EMT) and exhibit spindle cells. This sarcomatoid morphology is associated with very poor prognosis and a survival rate of 15–22% at 5 years (10).

In low grade RCC, treatment consists in partial or radical nephrectomy. Targeted- and immuno-therapies are the treatments of choice for inoperable metastatic RCC (11). Hanahan and Weinberg have proposed 10 organizing principles, called hallmarks, which are causative for tumor development and spread. Targeting one or, better, several hallmarks is thought to increase efficacy of anti-tumor therapies (12).

The tumor stroma is composed of cells (fibroblasts, mesenchymal stroma cells, pericytes, immune cells, vascular and lymphatic endothelial cells…) and extracellular matrix (ECM) (13). In RCC, several filamentous collagens are expressed and include type I (Col 1) and type III (Col 3) collagen. These are present in about half of the tumors, the remaining are represented by type V, VI, and XI collagen (14–16). The organization of the collagen fibers depends on the RCC grade. In high grade (Fuhrman grade IV) tumors fibers are aligned and the density is greater than in low grade tumors (17). Fibronectin 1 (FN1) or its alternative splicing variant EDA-FN are widely distributed in the RCC stroma (14, 16). RCC cells expressed FN1 and silencing its expression inhibits cell proliferation and invasion *in vitro* (18). Other components are derived from the basement membrane and include laminins (LNα1, β1-2, and γ1), collagen type IV (α1-2 chains), entactin (nidogen-1), tenascin-C, periostin and heparin-sulfate proteoglycans (HSPG) (14, 15, 19–21). ECM remodeling involves metalloproteinases (MMPs, mainly MMP-2, and 9) and cleavage of HSPGs by heparanase. All of these enzymes are increased in many metastatic cancers (22, 23).

In the present study, we analyzed the role of different ECM molecules (i.e., Col 1, FN1) and a mixed basement membrane components (Matrigel) in the phenotypic modulation of RCC cells.

## Materials and Methods

### *In silico* Analysis of RCC Patient Survival in The Human Protein Atlas

The impact of high protein expression on the survival of RCC patients was analyzed using the Pathology Atlas from The Human Protein Atlas (24). The Human Protein Atlas used transcriptomic data from TCGA. For RCC, data were available for 877 patients, 528 ccRCC patients and 285 pRCC patients. Overall survival was analyzed using Kaplan-Myer plots.

### RCC Cell Lines and ECM Used

The human 786-O cell line is derived from ccRCC mutated on the VHL gene (25). The human Caki-2 cell line was first classified as a ccRCC cell line. The VHL gene mutation status of this cell line is not well-defined but HIF1α and HIF2α are expressed (26). Caki-2 cells injected in mouse immunodeficient kidney develop in tumors resembling more pRCC (27). The Renca cell is a non VHL mutated ccRCC cell line derived from a spontaneous tumor in a BalbC mouse (28).

Rat tail Col 1 was obtained from Corning, bovine FN1 from Sigma Aldrich and Matrigel from Corning. Matrigel is a soluble basement membrane extract of murine Engelbreth-Holms-Swarm sarcoma tumor composed of LN, collagen IV, entactin, and HSPG where growth factors can be bind.

### Cell Culture

The mouse Renca and the human 786-O and Caki-2 cell lines were cultured in complete medium (RPMI complemented with 10% Fetal Bovine Serum (FBS); 100 U/ml penicillin and 100 μg/ml streptomycin) at 37°C and 5% CO_2_ in a humidified incubator.

For cell stimulation, dishes were coated with 400 μg/ml of Col 1, 5 μg/ml of FN1, or 33 μg/ml of Matrigel for 1 h at 37°C. Dishes were washes 3 times with PBS (Phosphate Buffer Saline) and used immediately.

### Cell Immunolabeling

RCC cells were cultured for 24 h on glass coverslips coated or not with the different ECMs, then fixed 10 min with 4% paraformaldehyde. Coverslips were blocked 1 h with 5% BSA (Bovine Serum Albumin) and 0.3% Triton X100 in PBS and incubated overnight at 4°C with mouse anti-β-catenin antibody diluted 1/800 (Cell Signaling Technology) in incubation buffer (1% BSA and 0.3% Triton X100 in PBS). After 3 washes in PBS, coverslips were incubated 1 h with appropriate FITC-conjugated secondary antibody, Alexa 556-conjugated Phalloïdin (1/500 dilution, FluoProbes) and DAPI (1 μg/ml, FluoProbes) in incubation buffer. After 3 washes in PBS, coverslips were mounted for microscope observation using Fluoromount G (Interchim). Cells were observed and pictures taken under a Nikon microscope.

### Cell Proliferation

Ten thousand (786-O and Caki-2) or 20,000 (Renca) cells in 500 μl of complete medium were cultured for 72 h in a 24 well plate coated or not with the different ECMs. Cells were trypsinized and counted using a Coulter Particle Counter (Beckman Coulter France).

### Cell Adhesion

Cell adhesion assay was performed as previously described with minor modifications (29). Briefly, cells were quickly trypsinized and washed 5 times in adhesion buffer (RPMI containing 0.1% BSA). Cells were counted, the concentration adjusted to 50,000 cells in 500 μl of adhesion buffer and leave 1 h at 37°C. Five hundred microliter of cells were deposed in a 24 well plate previously coated and blocked 30 min with 500 μl of adhesion buffer. After 1 h of incubation, dishes were washed 3 times with RPMI. Adherent cells were fixed with 4% paraformaldehyde for 10 min and nuclei were labeled 10 min with DAPI (2 μg/ml). Five pictures per dish were taken using a Zoe Fluorescent Cell Imager (Biorad). Nuclei were counted using Image J software.

### Cell Migration/Invasion Assays

Cell migration was assessed in two different assays, a scratch-wound assay and a Transwell assay.

For the scratch-wound assay 60,000 cells in 100 μl of complete medium were cultivated in 96-well plate ImageLock dishes coated or not with the different ECMs. When cells were at confluence the wound was performed using a Wound Maker (Essen BioScience), the cells were washed with PBS and 100 μl of complete medium were added. Cell migration was followed for 24 h using an IncuCyte system (Essen BioScience).

In the Transwell assay, 8 μm pore diameter inserts in 24-well plates (BD Falcon) were used and coated or not with the different ECMs. Twenty five thousand RCC cells in 500 μl of RPMI medium were put inside the insert. One milliliter of complete medium was used as chemoattractant. After 16 h at 37°C, cells were fixed with 4% paraformaldehyde for 20 min. After intensive PBS washes, non-migrating cells were removed. Nuclei from migrating cells were labeled with 1 μg/ml of DAPI for 20 min. After three washes, 5 photos/insert were taken and migrated cells counted with Image J software.

### Gelatin Zymography

The effect of ECMs on MMP-2 and MMP-9 activity was assessed by gelatin zymography on Renca, 786-O and Caki-2 cell lines. For this, 400,000 cells (Renca) or 300,000 cells (786-O and Caki-2) were cultured in complete medium for 24 h in 6 cm diameter petri dish coated or not with the different ECMs. Then, cells were cultured in fresh RPMI media without FBS for 24 h. Supernatants were harvested and centrifuged 10 min at 13,000 rpm. Thirty microliter of each sample in non-reducing loading buffer were loaded in 10% SDS-PAGE containing 0, 2% porcine gelatin (Sigma) and run at 100 V. Subsequently, the gel was rinsed with 2,5% Triton X-100 before being washed 4 times 15 min in 2,5% Triton X-100 at room temperature. After, the gel was rinsed with a revealing solution allowing enzymatic activity (50 mM Tris-HCl pH 7.4, 0.2 M NaCl, 10 mM CaCl_2_) before being incubated in this solution during 48 h at 37°C under agitation. Finally, the gel was stained with 0.5% Coomassie blue solution (0.5% Coomassie blue, 5% methanol, 10% acetic acid) and treated with destaining solution (30% ethanol, 10% acetic acid) until the appearance of clear bands. The gel was photographed and active MMP-2 and MMP-9 were quantified with image J software.

### RT-qPCR

Four thousand (Renca) or 300,000 (786-O and Caki-2) cells were cultivated in a 6 cm diameter dish coated or not with the different ECMs for 24 h. Total RNA was extracted using TRI Reagent (Molecular Research Center) following the manufacturer's instructions and quantified by spectrophotometry (Nanodrop, DS-11 DeNovix). Complementary DNA (cDNA) was synthesized with random primers from 500 ng of total RNA using reverse transcription kit (ThermoFisher Scientific) according to the manufacturer's instructions. The quantitative PCR was performed in duplicate on a CFX96 Real-Time System (C1000 Touch Thermal Cycler, Biorad) using TB Green Premix Ex Taq, Bulk (TaKaRa). The cycling parameters included 39 cycles of denaturation at 95°C for 30 s and annealing-elongation at 60°C for 30 s. Sequence specific primers (eurofins) designed and/or used to assess the mRNA expression of target genes are summarized in Table 1, and GAPDH was used as a housekeeping gene standard.

**Table 1:** Primers used in q-PCR analysis.

**Target**	**Human sequences**	**Mouse sequences**
MMP-2	GGAGACAAGTTCTGGAGATACAATG	GGAGACAAGTTCTGGAGATACAATG
	TTTGGTTCTCCAGCTTCAGGTA	TTTGGTTCTCCAGCTTCAGGTA
MMP-9	GTTCCCGGAGTGAGTTGAAC	CGTGTCTGGAGATTCGACTTGA
	TTTACATGGCACTGCCAAAGC	TGGAAACTCACACGCCAGAA
MMP-14	ACTGCCAAGCCACCCTAAGA	GCCCTCTGTCCCAGATAAGC
	CTGAGCAACGAAGACCCTCTCT	CCAGAACCATCGCTCCTTGA
Heparanase	ACTTCTTCACCCAGGAGCCG	AGTTTTACACCAAGCGGCCGC
	AGGTACGCAGGAGACAAGCC	GTATGCAGGAGATAAGCCTCTAG
Zeb1	TTACACCTTTGCATACAGAACCC	GCTGGCAAGACAACGTGAAAG
	TTTACGATTACACCCAGACTGC	GCCTCAGGATAAATGACGGC
Zeb2	GGAGACGAGTCCAGCTAGTGT	ATTGCACATCAGACTTTGAGGAA
	CCACTCCACCCTCCCTTATTTC	ATAATGGCCGTGTCGCTTCG
Snail 1	TCGGAAGCCTAACTACAGCGA	CACACGCTGCCTTGTGTCT
	AGATGAGCATTGGCAGCGAG	GGTCAGCAAAAGCACGGTT
Snail 2	CGAACTGGACACACATACAGTG	TGGTCAAGAAACATTTCAACGCC
	CTGAGGATCTCTGGTTGTGGT	GGTGAGGATCTCTGGTTTTGGTA
GAPDH	CAAGGAGTAAGACCCCTGGA	TGCCCCCATGTTTGTGATG
	AGGGGAGATTCAGTGTGGTG	TGTGGTCATGAGCCCTTCC

### Sensibility to Drugs-MTS Cell Viability Assay

Two thousand Renca, 1,500 786-O or 1,000 Caki-2 cells in 100 μl of complete medium were cultured for 24 h on 96-well plates coated or not with the different ECMs. After removing the media, 100 μl of new media containing increasing concentrations of Pazopanib and Sorafenib [0.03 to 30 μM and 0.01 to 10 μM, respectively, for Pazopanib and Sorafenib (Enzo Life Sciences)] were added. After 24 h of treatment, 10 μL/well of MTS (Promega) were added to the cells for 2 h at 37°C. Finally, the optical density was read at 490 nm using a microplate reader (CLARIOstar^*Plus*^). The results are expressed as (OD experiment—OD blank) where OD blank represent the optical density of the wells with media alone.

### Statistical Analysis

Statistical analysis was performed using the GraphPad Prism 5 software. Comparisons were performed with One Way ANOVA analysis of variance, followed by Dunnett's Multiple Comparison Test. ⋆, *P* < 0.05; ⋆⋆, *P* < 0.01; ⋆⋆⋆, *P* < 0.001.

## Results

### Kidney Extracellular Matrix and 5 Years Patient Survival

We first investigated whether expression of the main components of the ECM is correlated to kidney cancer aggressiveness in The Protein Atlas dataset and analyzed the 5-years survival of kidney cancer patients. Col 1 (1A1 or 1A2) or FN1 expressions correlated with reduced survival of ccRCC and pRCC patients (Table 2). In contrast, ccRCC patients with higher expression of LNα1, LNγ1, Col 4A2, or entactin (Nidogen 1) have a better survival rate However for pRCC, survival was reduced in this case (Table 2). In addition, LNβ1, expression is correlated with reduced survival in ccRCC. Furthermore, patients with high Col 4A3 expression have better survival rate in ccRCC (Table 2). For HSPG, high expression of transmembrane receptors of perlecan or syndecan 2, is correlated with better survival in ccRCC patients (Table 2). For GPI anchored HSPG, ccRCC patients with high expression of glypican 1, 2, 3, and 5 have lower survival rate, whereas patients with high expression of glypican 4 and 6 have higher survival rate (Table 2).

**Table 2:** Kidney extracellular matrix and 5 years patient survival.

	**All renal cancer**	**Kidney renal clear cell carcinoma: ccRCC**	**Kidney renal papillary cell carcinoma: pRCC**
Col 1A1	– P (7.2.10^−12^)	– (4.10^−4^)	– (8.1.10^−10^)
Col 1A2	– P (1.5.10^−9^)	– (0.0078)	– (1.7.10^−7^)
FN1	– P (3.4.10^−8^)	– (0.0044)	– (1.4.10^−7^)
LNα1	NS (0.19)	+ (0.02)	– (0.026)
LNβ1	+ (0.029)	– (0.0021)	NS (0.27)
LNγ1	– (0.0019)	+ (0.0038)	– (2.5.10^−5^)
LNβ2	+ (0.0012)	+ (1.4.10^−7^)	N/A
Col 4A2	– P (3.5.10^−5^)	+ (0.004)	– (0.0036)
Col 4A3	+ P (2.5.10^−7^)	+ (1.3.10^−5^)	NS (0.31)
Entactin (Nid1)	– P (5.4.10^−8^)	+ (0.006)	– (5.5.10^−7^)
HSPG2 (Perlecan)	+ (0.0012)	+ (3.5.10^−9^)	– (5.2.10^−4^)
Syndecan 1	NS (0.16)	NS (0.2)	NS (0.44)
Syndecan 2	+ (0.016)	+ (0.0014)	NS (0.54)
Syndecan 3	– P (1.7.10^−4^)	– (0.0036)	NS (0.15)
Syndecan 4	+ P (2.4.10^−7^)	NS (0.29)	+ (0.0024)
Glypican 1	NS (0.19)	– (3.3.10^−6^)	– (0.02)
Glypican 2	– (1.7.10^−4^)	– (2.9.10^−10^)	+ (0.041)
Glypican 3	– (0.0038)	– (0.008)	– (6.2.10^−4^)
Glypican 4	+ P (1.2.10^−5^)	+ (1.4.10^−5^)	N/A
Glypican 5	– (1.8.10^−6^)	– (0.0062)	– (0.0091)
Glypican 6	+ (0.0025)	+ (1.3.10^−6^)	+ (0.018)

*Data were obtained from The Human Protein Atlas dataset (https://www.proteinatlas.org) for the different ECM components in RCC, ccRCC, or in pRCC. +, high expression is favorable in renal cancer; -, high expression is unfavorable in renal cancer; NS, high expression has no specific effect on patient survival; in parentheses is found the P score 5 year survival value. N/A, non applicable; P, the ECM molecule is a pronostic factor for RCC*.

Some highly expressed ECM components (including Col 1A1, 1A2, 4A2, FN1, entactin, syndecan 3) are of bad prognosis. However, high Col 4A3, syndecan 4, and glypican 4 expression is of good prognosis in RCC (Table 2).

Altogether these results suggest a negative correlation between the expression of ECM components, that are not part of the basal lamina, and 5-years survival of ccRCC and pRCC patients. On the contrary, a positive correlation was observed between several basal lamina ECM components and ccRCC patient survival. For pRCC a negative correlation was observed in this case.

### Effect of the Different ECM Components on the Phenotype of RCC Cells

**Figure 1:**
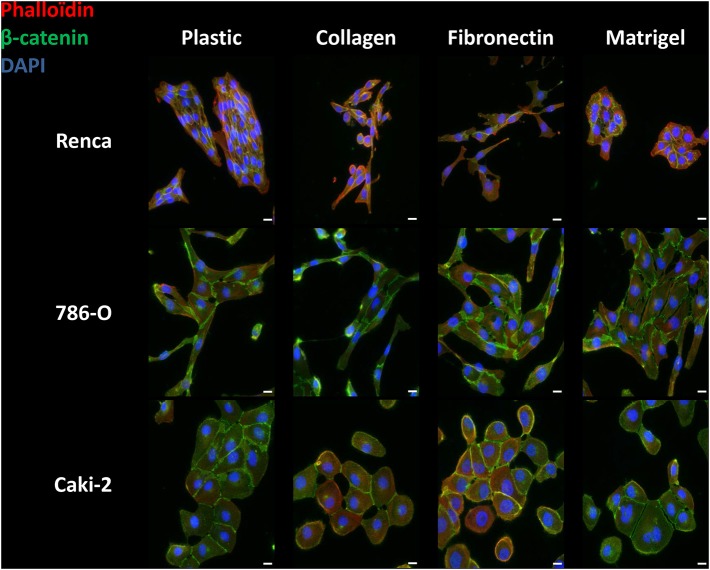
Effect of the different ECM components on the phenotype of RCC cells. Cells were cultured for 24 h on uncoated (Plastic) or Col 1, FN1 or Matrigel glass coverslips, fixed and stained with β-catenin antibody (green), with phalloïdin (filamentous actin, red), and with DAPI (nucleus, blue). Images were captured using a Nikon microscope. Bar: 20 μm. *N* = 3.

RCC cells were cultured for 24 h on the different ECM molecules and immunolabeled with anti-β-catenin antibody for visualization of cell-cell junctions and with phalloïdin for actin filament remodeling (Figure 1, Supplementary Figures 1–3).

On plastic and without any stimulation, Renca cells grew in clusters and exhibited cell to cell junctions. On Col 1 or FN1, Renca cell were more dissociated and fusiform with numerous membrane extensions. On Matrigel, cells grew in clusters and arrange themselves as acini. Cortical actin was not modified.

The Human RCC 786-O cell line cultured on plastic, Col 1 or FN1, acquired an elongated shape with cell-cell junctions. On Matrigel, cells were not elongated and grew in clusters. On all ECM components, only sparse cortical actin is observed. Furthermore, on Col 1, cortical actin was found at membrane extensions.

The Caki-2 RCC cell line was also tested. Caki-2 cells had a round shape and grew in clusters with β-catenin at cell-cell junctions but with little cortical actin, excepted when grown on FN1.

Taken together, different RCC cells adopted various phenotypic changes when cultured in the different ECMs.

### Effect of the Different ECM Components on RCC Cell Proliferation

**Figure 2:**
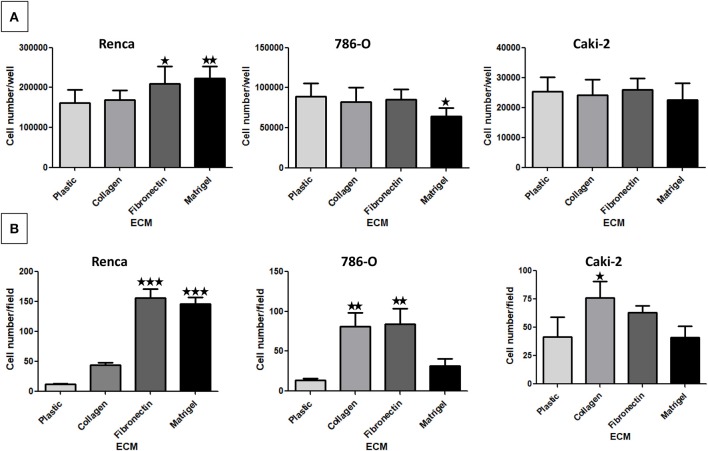
Effect of the different ECM components on RCC cell proliferation **(A)** and adhesion **(B)**. **(A)** Renca, 786-O, and Caki-2 cells were cultured on uncoated (Plastic) or Col 1, FN1 or Matrigel coated dishes for 72 h. Then cells were detached and counted. Renca, *N* = 4; 786-O and Caki-2, *N* = 3. **(B)** Renca, 786-O, and Caki-2 cells were allowed to adhere on plastic, Col 1, FN1, or Matrigel for 1 h, fixed and nuclei labeled. Five photos per well were taken and nuclei counted using ImageJ software. *N* = 3. ^⋆^*P* < 0.05; ^⋆⋆^*P* < 0.01; ^⋆⋆⋆^*P* < 0.001.

The impact of the ECM components on RCC cell proliferation was analyzed after 72 h (Figure 2A). We observed only a small increase in proliferation when Renca cells were cultured on FN1 or on Matrigel. On the other end, proliferation of 786-O cells was decreased to some extent when grown on Matrigel. No effect of the ECMs on Caki-2 proliferation was observed. Thus, ECM components had only a minor impact on the proliferation of RCC cells.

### Effect of the Different ECM Components on RCC Cell Adhesion

Cell adhesion to ECM is mainly mediated by integrins (30). Cell adhesion assays were performed on the different ECMs (Figure 2B). Renca cell adhesion was greatly increased on FN1 and on Matrigel. Adhesion of 786-O cells was increased on Col 1 and on FN1. On the contrary, adhesion of Caki-2 cells to Col 1 was only increased to a small extent. These results show that these RCC cell types present different adhesion profiles when cultured in the different ECMs.

### Effect of the Different ECM Components on RCC Cell Migration

**Figure 3:**
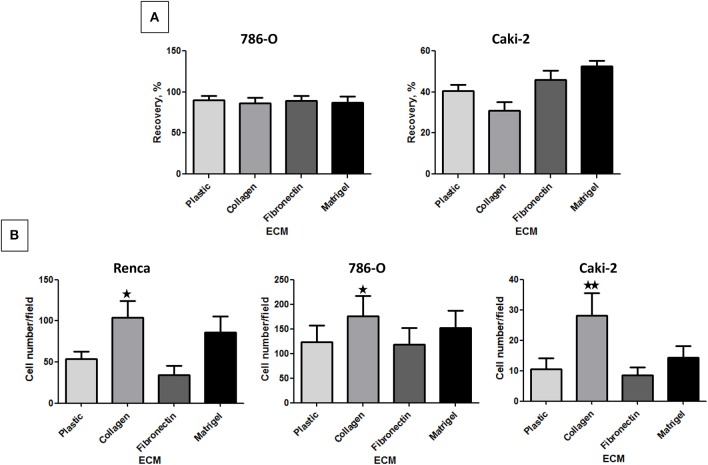
Effect of the different ECM components on RCC cell migration. **(A)** 786-O or Caki-2 cell migration was assessed in a scratch-wound assay with cells cultured on plastic, Col 1, FN1, or Matrigel. The kinetic of the cell migration into the wound was followed using an IncuCyte system (Essen BioScience). The percentage of wound closure was obtained after 24 h of cell migration, *N* = 4. **(B)** Renca, 786-O, or Caki-2 cell migration was assessed in a Transwell assay. Filters were uncoated (Plastic) or previously coated with Col 1, FN1, or Matrigel and FBS was used as chemoattractant. After 16 h, migrated cells were fixed and nuclei labeled. Five photos per well were taken and nuclei counted using ImageJ software. Renca: *N* = 4; 786-O: *N* = 5; Caki-2: *N* = 6. ^⋆^*P* < 0.05; ^⋆⋆^*P* < 0.01; ^⋆⋆⋆^*P* < 0.001.

We next performed a scratch wound assay. In this case, no significant difference in cell migration was observed for 786-O and Caki-2 cells when cultured on the different ECMs. Renca cells did not migrate efficiently in this assay (Figure 3A).

In the Transwell assay, cells are attracted to the lower side of the insert by serum present in the lower chamber. From the 3 cell lines, 786-O cells had a higher migrating capacity in absence of ECM. Col 1 was the only ECM, which significantly increased the migration of all 3 cell lines (Figure 3B).

### Effect of the Different ECM Components on Active Proteases and on Protease Expression

**Figure 4:**
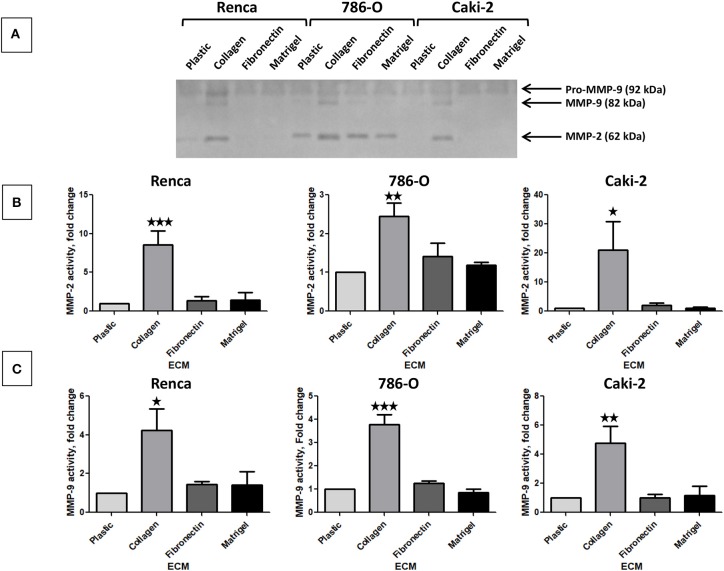
Effect of the different ECM components on active MMP-2 and MMP-9. **(A)** gelatin zymography was assessed on supernatants of RCC cells cultured on plastic, Col 1, FN1, or Matrigel for 24 h as indicated in materiel and methods. *N* = 4, a representative gel is presented. **(B)** quantification of active MMP-2 normalized to cell grown on plastic, *N* = 4. **(C)** quantification of active MMP-9 normalized to cell grown on plastic, *N* = 4. ^⋆^*P* < 0.05; ^⋆⋆^*P* < 0.01; ^⋆⋆⋆^*P* < 0.001.

The activity of secreted MMP-2 and MMP-9 was assessed by gelatin zymography using cell culture supernatants. In absence of ECM, the 3 cell lines expressed Pro-MMP-9 but active MMP-9 or MMP-2 were only detected in 786-O supernatant (Figure 4A). Col 1 was the only ECM molecule that increased MMP-9 and MMP-2 activity in the three cell lines. FN1 and Matrigel had no effect on MMP-9 or 2 activity (Figures 4A–C).

**Figure 5:**
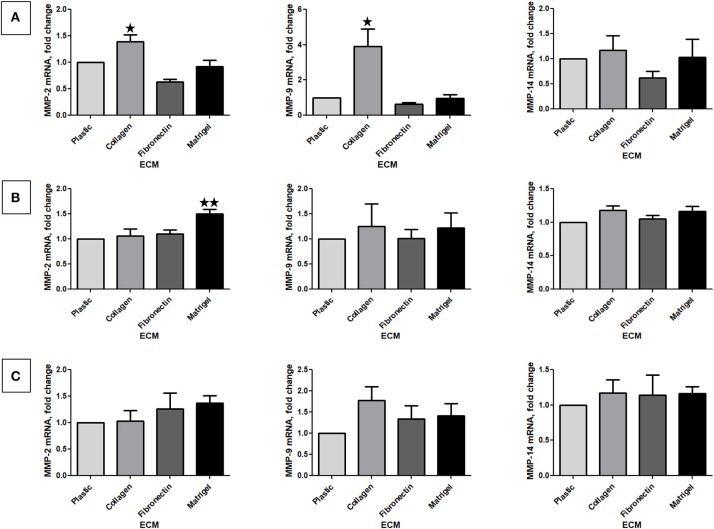
Effect of the different ECM components on MMP-2, 9, and 14 mRNA expression. Relative mRNA levels for MMP-2, 9, and 14 were assessed by RT-qPCR after 24 h of RCC cells cultured on plastic, Col 1, FN1, or Matrigel. **(A)** Renca cells, *N* = 3. **(B)** 786-O cells, *N* = 4. **(C)** Caki-2 cells, *N* = 4. ^⋆^*P* < 0.05; ^⋆⋆^*P* < 0.01; ^⋆⋆⋆^*P* < 0.001.

We next investigated MMP-2 and 9 mRNA by RT-qPCR analysis of RNA in RCC cells stimulated or not by the different ECM components. Because it is well-known that the activation of MMP-2 and, in some instance, of MMP-9 is MMP-14 (MT1-MMP)-dependent, we also measured MMP-14 mRNA expression (31, 32). Renca cells cultured on Col 1 exhibited an increase in MMP-2 and MMP-9 mRNA but not in MMP-14 mRNA expression (Figure 5A). This indicates that the increase MMP activity is mediated, at least in part, by an increase in MMP-2 or MMP-9 mRNA. 786-O and Caki-2 cells grown on the different ECMs did not showed changes in MMP mRNA expression, excepted for MMP-2 in 786-O cultured on Matrigel (Figures 5B,C).

Heparanase mRNA expression was quantified by RT-qPCR in RCC cells stimulated or not by the different ECM molecules. Using 2 different sets of primers, no heparanase expression was found in Renca cells. In contrast, heparanase mRNA was expressed in 786-O and Caki-2 cells but no modulation by the different ECMs was observed (Supplementary Figure 4).

### Effect of the Different ECM Components on the Expression of Transcription Factors Implicated in EMT

**Figure 6:**
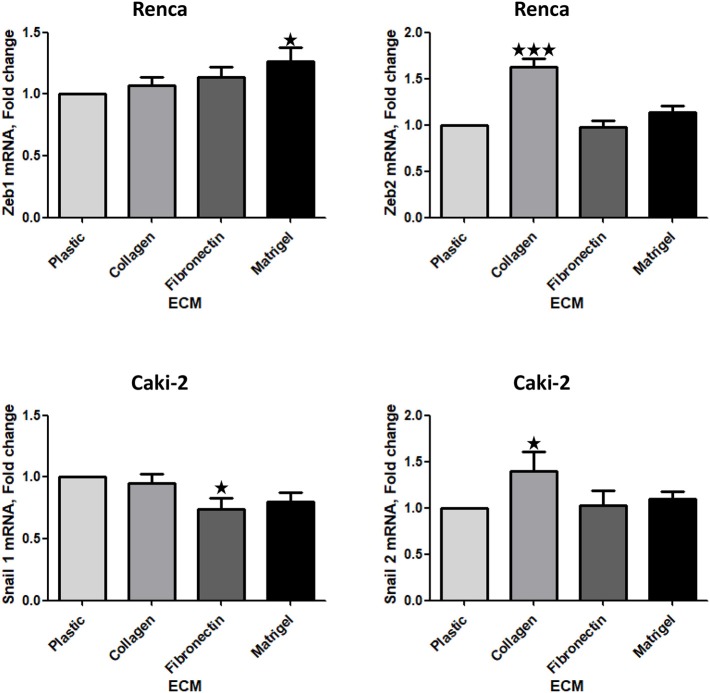
Effect of the different ECM components on the expression of transcription factors implicated in EMT. Relative mRNA levels for Zeb1, Zeb2, Snail 1, and Snail 2 were assessed by RT-qPCR after 24 h of RCC cells cultured on plastic, Col 1, FN1, or Matrigel. Upper panels, Renca cells, *N* = 7. Lower panels, Caki-2 cells, *N* = 5. ^⋆^*P* < 0.05; ^⋆⋆^*P* < 0.01; ^⋆⋆⋆^*P* < 0.001.

We next measured the expression of mRNA of several transcription factors associated with EMT. No difference in Zeb1, 2 or Snail 1, 2 mRNA was observed in 786-O cells cultured on the different ECMs (Supplementary Figures 5, 6). In contrast, in Renca cells, Zeb1 and Zeb2 mRNAs were upregulated, respectively, when cells were seeded on Matrigel or Col 1 (Figure 6). In Caki-2, Col 1 increased Snail 2 mRNA expression. In opposite, when cells were seeded on FN1 a small but significant downregulation of Snail 1 mRNA was observed (Figure 6). These results indicate that EMT-associated transcription factors are modulated by ECM components.

### Effect of ECM on Drug Sensitivity

**Figure 7:**
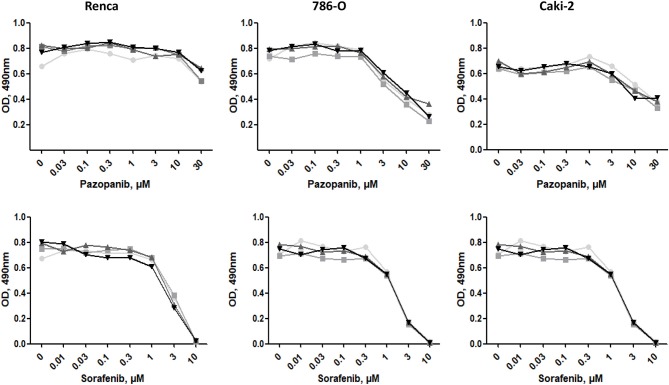
Effect of ECM on drug sensitivity. RCC cells were cultured on plastic 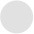, Col 1 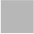, FN1 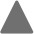, or Matrigel 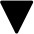 for 24 h. Then, cells were treated with increasing concentrations of Pazopanib or Sorafenib for 24 h. Cell viabilities were assessed with MTS.

We next investigated whether the different ECMs may have a protective effect when cells are exposed to tyrosine-kinase inhibitors (TKI) such as Sorafenib or Pazopanib, two TKI used in clinic. Cells, seeded on the different matrices for 24 h, were incubated with increasing concentrations of Sorafenib or Pazopanib for another 24 h and cell survival was measured with MTS. As shown in Figure 7, ECM components had no effect on the chemosensitivity of the three RCC cell lines to this two TKI.

## Discussion

Analysis of the 5-year patient survival in the The Human Protein Atlas reveals a negative correlation between ccRCC and pRCC and high Col 1 expression. This is in agreement with other cancers where collagens are factors of bad prognosis, e.g., Col A1 in colorectal and breast cancers (33).

The different RCC cells adopted various phenotypic changes when cultured in the different ECMs. Cell morphology is mainly dependent on cell signaling and on ECM stiffness (34). The tumor cells, used in this study are genetically different (VHL/HIF2α) and expressed different integrins and ECM receptors. Consequently, these cells respond differently to different ECM.

In most cancers, collagen expression is related to cell migration, invasion and metastasis. Col 1 increases migration of three RCC cell lines and stimulate MMP-2 and 9 activity, two metalloproteinases implicated in cancer cell invasion and metastasis formation (22). Moreover, Col 1 increases the expression of Zeb2 in Renca cells or Snail 2 in Caki-2 cells, two transcription factors implicated in EMT. However, in 786-O cells, no increase in EMT-related transcription factors was observed. VHL is implicated in the inhibition of EMT (25) and 786-O is a VHL negative cell line. In the Renca cell line, CRISPR inactivation of VHL induced EMT (28). We postulate that, in the VHL negative 786-O, the amount of the transcription factors implicated in EMT is already too high to be regulated by Col 1. Alternatively in this cell line, Col 1 increases the expression of other transcription factors such as Sox4 or Twist 1 (35).

Col 1 binding to their membrane receptors can directly activate EMT. Col 1 can bind to at least two types of receptors, integrins and the discoidin domain receptors, DDR1 and DDR2. Integrins and DDRs are potent EMT inducers. Col 1 binding to integrins can activate AKT and GSK3β and, in turn, directly EMT (36). DDRs exhibit tyrosine kinase activity and are implicated in cell proliferation, migration, invasion and EMT (37). Alternatively, as in lung cancer, the role of Col 1 in EMT is indirect and can be mediated via TGFβ3 expression (38). The role of DDRs in RCC development and EMT is under investigation.

High FN1 concentration correlated with poor survival in RCC patients. RCC cells exhibited increased adhesion on FN1 compare to plastic, suggesting that integrins such as αvβ3 or α5β1 are implicated (39). However, we did not evidence an effect on RCC cell migration and MMP activity. Poor survival may be alternatively related to a role of FN1 in the tumor microenvironment. It is well-known that FN1 is an RGD-motif containing protein able to bind the αvβ3 integrins present at the cell surface of endothelial cells and induces angiogenesis. Targeting angiogenesis by inhibiting FN1 binding to αvβ3 inhibits tumor angiogenesis and tumor growth in teratocarcinoma and in the ccRCC cell line 786-O (40).

With the exception of LNβ1 and glypican 1-3, 5, all the other basement membrane proteins found in RCC, e.g., LNα1, γ1, β2, Col IV, entactin, syndecan, glypican 4, and 6 and perlecan, are good prognostic factors for ccRCC but bad prognostic factors for pRCC. Matrigel, a basement membrane matrix derived from a Engelbreth-Holms-Swarm sarcoma which contains LN, Col IV, entactin and HSPG, had no significant effect on the phenotype of 786-O and Caki-2 cells. In Renca cells, Matrigel stimulated slightly cell proliferation, as well as MMP-2 and Zeb1 mRNA expression but without interfering significantly with cell migration.

Heparanase, an enzyme implicated in the degradation of HSPG is found upregulated in many cancers (23) such as in advanced-stage RCC (2, 41). Little is known about heparanase's transcription regulation, only estrogen receptor activity or inflammatory mediators TNFα or IFNα were found to increase heparanase mRNA (42). In our study, Col 1, FN1 or other main components of the basement membranes, were unable to modulate heparanase expression.

It is well-known that the ECM is able to modulate the sensitivity of cells to anti-cancer drugs and that density and stiffness can inhibit accessibility of drugs to the tumor. This was particularly well-study in breast cancers (43) but little is known in RCC. *In vitro* experiments did not reveal an effect of ECM components on the sensitivity to Pazopanib and Sorafenib, two TKI commonly used in clinic. In breast cancers, ECM stiffness creates a protective barrier that reduces the accessibility to TKI (43). In our experiments, low ECM concentrations were used, which results in insufficient stiffness to create a barrier to drug accessibility.

Tumor cells adhered differently on the ECMs, indicating that different RCC cell lines do not express the same integrins. Renca cell adhesion to Col 1 is low, suggesting that α1, 2, 10, or 11/β1 are expressed at low level. On the contrary, 786-O and Caki-2 are likely to express high level of these integrins. All RCC cells adhered to FN1 suggesting a strong expression of FN1- specific integrins, such as αvβ5. Only Renca cells adhere well to Matrigel, which is in favor of high expression of LN-specific integrins such as α6β1 (39).

Taken together, we show in this study that the effect of ECM components on various RCC cell lines is heterogenous varying according to RCC cell type and matrix with Col 1 being the main enhancer of tumor cell invasion, of MMP-2 and 9 activity and consequently to metastasis.

## Data Availability Statement

All datasets generated for this study are included in the article/Supplementary Material.

## Author Contributions

SM, SC, and WS contributed to data acquisition and statistical analysis. SM prepared the manuscript. AB and PA drafted this manuscript. PA supervised the study. All authors read and approved the final manuscript.

## Conflict of Interest

The authors declare that the research was conducted in the absence of any commercial or financial relationships that could be construed as a potential conflict of interest.

## Supplementary Material

Supplementary Figure 1 Effect of the different ECM components on the phenotype of Renca cells. Cells were cultured for 24 h on uncoated (Plastic) or Col 1, FN1, or Matrigel glass coverslips, fixed and stained with β-catenin antibody or with phalloïdin (filamentous actin). Bar: 20 μm. *N* = 3.

Supplementary Figure 2 Effect of the different ECM components on the phenotype of 786-O cells. Cells were cultured for 24 h on uncoated (Plastic) or Col 1, FN1, or Matrigel glass coverslips, fixed and stained with β-catenin antibody or with phalloïdin (filamentous actin). Bar: 20 μm. *N* = 3.

Supplementary Figure 3 Effect of the different ECM components on the phenotype of Caki-2 cells. Cells were cultured for 24 h on uncoated (Plastic) or Col 1, FN1, or Matrigel glass coverslips, fixed and stained with β-catenin antibody or with phalloïdin (filamentous actin). Bar: 20 μm. *N* = 3.

Supplementary Figure 4 Effect of the different ECM components on heparanase mRNA expression. Relative mRNA levels for heparanase were assessed by RT-qPCR after 24 h of RCC cells cultured on plastic, Col 1, FN1, or Matrigel. No heparanase mRNA were detected in Renca cells. *N* = 4.

Supplementary Figure 5 Effect of the different ECM components on the expression of Zeb1 and 2, two transcription factors implicated in EMT. Relative mRNA levels for Zeb1 **(A)** and Zeb2 **(B)** were assessed by RT-qPCR after 24 h of RCC cells cultured on plastic, Col 1, FN1, or Matrigel. 786-O cells: *N* = 4. Caki-2 cells: *N* = 5.

Supplementary Figure 6 Effect of the different ECM components on the expression of Snail 1 and 2, two transcription factors implicated in EMT. Relative mRNA levels for Snail 1 **(A)** and Snail 2 **(B)** were assessed by RT-qPCR after 24 h of RCC cells cultured on plastic, Col 1, FN1 or Matrigel. Renca cells, *N* = 7. 786-O cells: *N* = 4.

## References

[1] Mancini V Battaglia M Ditonno P Palazzo S Lastilla G Montironi R. Current insights in renal cell cancer pathology. Urol Oncol. (2008) 26:225–38. 10.1016/j.urolonc.2007.05.017 18452811

[2] Mikami S Oya M Mizuno R Kosaka T Ishida M Kuroda N. Recent advances in renal cell carcinoma from a pathological point of view. Pathol Int. (2016) 66:481–90. 10.1111/pin.12433 27461942

[3] Muglia VF Prando A. Renal cell carcinoma: histological classification and correlation with imaging findings. Radiol Bras. (2015) 48:166–74. 10.1590/0100-3984.2013.1927 26185343 PMC4492569

[4] Lopez-Beltran A Carrasco JC Cheng L Scarpelli M Kirkali Z Montironi R. 2009 update on the classification of renal epithelial tumors in adults. Int J Urol. (2009) 16:432–43. 10.1111/j.1442-2042.2009.02302.x 19453547

[5] Meléndez-Rodríguez F Roche O Sanchez-Prieto R Aragones J. Hypoxia-inducible factor 2-dependent pathways driving von hippel-lindau-deficient renal cancer. Front Oncol. (2018) 8:214. 10.3389/fonc.2018.00214 29938199 PMC6002531

[6] Kim JS Song KS Yu IJ. Multiwall carbon nanotube-induced DNA damage and cytotoxicity in male human peripheral blood lymphocytes. Int J Toxicol. (2015) 35:27–37. 10.1177/1091581815598749 26268766

[7] Delahunt B Eble JN. Papillary renal cell carcinoma: a clinicopathologic and immunohistochemical study of 105 tumors. Mod Pathol. (1997) 10:537–44. 9195569

[8] Fuhrman SA Lasky LC Limas C. Prognostic significance of morphologic parameters in renal cell carcinoma. Am J Surg Pathol. (1982) 6:655–63. 10.1097/00000478-198210000-00007 7180965

[9] Bretheau D Lechevallier E de Fromont M Sault MC Rampal M Coulange C. Prognostic value of nuclear grade of renal cell carcinoma. Cancer. (1995) 76:2543–9. 10.1002/1097-0142(19951215)76:12<2543::AID-CNCR2820761221>3.0.CO;2-S 8625083

[10] Delahunt B Eble JN Egevad L Samaratunga H Grading of renal cell carcinoma. Histopathology. (2019) 74:4–17. 10.1111/his.13735 30565310

[11] Bæk Møller N Budolfsen C Grimm D Krüger M Infanger M Wehland M Drug-induced hypertension caused by multikinase inhibitors (Sorafenib, Sunitinib, Lenvatinib and Axitinib) in renal cell carcinoma treatment. Int J Mol Sci. (2019) 20:4712 10.3390/ijms20194712 PMC6801695 31547602

[12] Hanahan D Weinberg RA. Hallmarks of cancer: the next generation. Cell. (2011) 144:646–74. 10.1016/j.cell.2011.02.013 21376230

[13] Roma-Rodrigues C Mendes R Baptista PV Fernandes AR. Targeting tumor microenvironment for cancer therapy. Int J Mol Sci. (2019) 20:840. 10.3390/ijms20040840 30781344 PMC6413095

[14] Lohi J Leivo I Oivula J Lehto VP Virtanen I. Extracellular matrix in renal cell carcinomas. Histol Histopathol. (1998) 13:785–96. 9690136 10.14670/HH-13.785

[15] Droz D Patey N Paraf F Chrétien Y Gogusev J. Composition of extracellular matrix and distribution of cell adhesion molecules in renal cell tumors. Lab Invest. (1994) 71:710–8. 7526040

[16] Boguslawska J Kedzierska H Poplawski P Rybicka B Tanski Z Piekielko-Witkowska A. Expression of genes involved in cellular adhesion and extracellular matrix remodeling correlates with poor survival of patients with renal cancer. J Urol. (2016) 195:1892–902. 10.1016/j.juro.2015.11.050 26631499

[17] Best SL Liu Y Keikhosravi A Drifka CR Woo KM Mehta GS. Collagen organization of renal cell carcinoma differs between low and high grade tumors. BMC Cancer. (2019) 19:490. 10.1186/s12885-019-5708-z 31122202 PMC6533752

[18] Ou YC Li JR Wang JD Chang CY Wu CC Chen WY. Fibronectin promotes cell growth and migration in human renal cell carcinoma cells. Int J Mol Sci. (2019) 20:2792. 10.3390/ijms20112792 31181623 PMC6600362

[19] Bakhtyar N Wong N Kapoor A Cutz JC Hill B Ghert M. Clear cell renal cell carcinoma induces fibroblast-mediated production of stromal periostin. Eur J Cancer. (2013) 49:3537–46. 10.1016/j.ejca.2013.06.032 23896380

[20] Lohi J Korhonen M Leivo I Kangas L Tani T Kalluri R. Expression of type IV collagen α1(IV)-α6(IV) polypeptides in normal and developing human kidney and in renal cell carcinomas and oncocytomas. Int J Cancer. (1997) 72:43–9. 10.1002/(SICI)1097-0215(19970703)72:1<43::AID-IJC6>3.0.CO;2-4 9212221

[21] Lohi J Tani T Leivo I Linnala A Kangas L Burgeson RE. Expression of laminin in renal-cell carcinomas, renal-cell carcinoma cell lines and xenografts in nude mice. Int J Cancer. (1996) 68:364–71. 10.1002/(SICI)1097-0215(19961104)68:3<364::AID-IJC15>3.0.CO;2-8 8903479

[22] Winer A Adams S Mignatti P. Matrix metalloproteinase inhibitors in cancer therapy: turning past failures into future successes. Mol Cancer Ther. (2018) 17:1147–55. 10.1158/1535-7163.MCT-17-0646 29735645 PMC5984693

[23] Masola V Zaza G Gambaro G Franchi M Onisto M. Role of heparanase in tumor progression: molecular aspects and therapeutic options. Semin Cancer Biol. (2019) 62:86–98. 10.1016/j.semcancer.2019.07.014 31348993

[24] Uhlen M Zhang C Lee S Sjöstedt E Fagerberg L Bidkhori G. A pathology atlas of the human cancer transcriptome. Science. (2017) 357:660. 10.1126/science.aan2507 28818916

[25] Iliopoulos O Kibel A Gray S Kaelin WG. Tumour suppression by the human von Hippel-Lindau gene product. Nat Med. (1995) 1:822–6. 10.1038/nm0895-822 7585187

[26] Shinojima T Oya M Takayanagi A Mizuno R Shimizu N Murai M. Renal cancer cells lacking hypoxia inducible factor (HIF)-1alpha expression maintain vascular endothelial growth factor expression through HIF-2alpha. Carcinogenesis. (2007) 28:529–36. 10.1093/carcin/bgl143 16920734

[27] Brodaczewska KK Szczylik C Fiedorowicz M Porta C Czarnecka AM. Choosing the right cell line for renal cell cancer research. Mol Cancer. (2016) 15:83. 10.1186/s12943-016-0565-8 27993170 PMC5168717

[28] Schokrpur S Hu J Moughon DL Liu P Lin LC Hermann K. CRISPR-mediated VHL knockout generates an improved model for metastatic renal cell carcinoma. Sci Rep. (2016) 6:29032. 10.1038/srep29032 27358011 PMC4928183

[29] Croissant C Tuariihionoa A Bacou M Souleyreau W Sala M Henriet E. DDR1 and DDR2 physical interaction leads to signaling interconnection but with possible distinct functions. Cell Adh Migr. (2018) 12:324–34. 10.1080/19336918.2018.1460012 29616590 PMC6363034

[30] Bachmann M Kukkurainen S Hytönen VP Wehrle-Haller B. Cell Adhesion by Integrins. Physiol Rev. (2019) 99:1655–99. 10.1152/physrev.00036.2018 31313981

[31] Toth M Chvyrkova I Bernardo MM Hernandez-Barrantes S Fridman R. Pro-MMP-9 activation by the MT1-MMP/MMP-2 axis and MMP-3: role of TIMP-2 and plasma membranes. Biochem Biophys Res Commun. (2003) 308:386–95. 10.1016/S0006-291X(03)01405-0 12901881

[32] Hernandez-Barrantes S Toth M Bernardo MM Yurkova M Gervasi DC Raz Y. Binding of active (57 kDa) membrane type 1-matrix metalloproteinase (MT1-MMP) to tissue inhibitor of metalloproteinase (TIMP)-2 regulates MT1-MMP processing and pro-MMP-2 activation. J Biol Chem. (2000) 275:12080–9. 10.1074/jbc.275.16.12080 10766841

[33] Xu S Xu H Wang W Li S Li H Li T. The role of collagen in cancer: from bench to bedside. J Transl Med. (2019) 17:309. 10.1186/s12967-019-2058-1 31521169 PMC6744664

[34] Wolfenson H Yang B Sheetz MP. Steps in mechanotransduction pathways that control cell morphology. In: Nelson MT, Walsh K, editors. Annual Review of Physiology, Vol. 81. Palo Alto, CA: Annual Reviews (2019). p. 585–605. 10.1146/annurev-physiol-021317-121245 PMC7476682 30403543

[35] Pastushenko I Blanpain C. EMT transition states during tumor progression and metastasis. Trends Cell Biol. (2019) 29:212–26. 10.1016/j.tcb.2018.12.001 30594349

[36] Lamouille S Xu J Derynck R. Molecular mechanisms of epithelial-mesenchymal transition. Nat Rev Mol Cell Biol. (2014) 15:178–96. 10.1038/nrm3758 24556840 PMC4240281

[37] Valiathan RR Marco M Leitinger B Kleer CG Fridman R. Discoidin domain receptor tyrosine kinases: new players in cancer progression. Cancer Metastasis Rev. (2012) 31:295–321. 10.1007/s10555-012-9346-z 22366781 PMC3351584

[38] Shintani Y Maeda M Chaika N Johnson KR Wheelock MJ. Collagen I promotes epithelial-to-mesenchymal transition in lung cancer cells via transforming growth factor-beta signaling. Am J Respir Cell Mol Biol. (2008) 38:95–104. 10.1165/rcmb.2007-0071OC 17673689 PMC2176131

[39] Humphries JD Byron A Humphries MJ. Integrin ligands at a glance. J Cell Sci. (2006) 119:3901–3. 10.1242/jcs.03098 16988024 PMC3380273

[40] Eikesdal HP Sugimoto H Birrane G Maeshima Y Cooke VG Kieran M. Identification of amino acids essential for the antiangiogenic activity of tumstatin and its use in combination antitumor activity. Proc Natl Acad Sci USA. (2008) 105:15040–5. 10.1073/pnas.0807055105 18818312 PMC2567489

[41] Mikami S Oya M Shimoda M Mizuno R Ishida M Kosaka T. Expression of heparanase in renal cell carcinomas: implications for tumor invasion and prognosis. Clin Cancer Res. (2008) 14:6055–61. 10.1158/1078-0432.CCR-08-0750 18809970

[42] Ilan N Elkin M Vlodavsky I. Regulation, function and clinical significance of heparanase in cancer metastasis and angiogenesis. Int J Biochem Cell Biol. (2006) 38:2018–39. 10.1016/j.biocel.2006.06.004 16901744

[43] Fleming JM Yeyeodu ST McLaughlin A Schuman D Taylor DK. *In situ* drug delivery to breast cancer-associated extracellular matrix. ACS Chem Biol. (2018) 13:2825–40. 10.1021/acschembio.8b00396 30183254

