# Supplementary figures and images for: Impact of Extracellular Matrix Components to Renal Cell Carcinoma Behavior
Source: Front Oncol. 2020 Apr 28;10:625. doi: 10.3389/fonc.2020.00625 (PMC7198871; doi:10.3389/fonc.2020.00625)

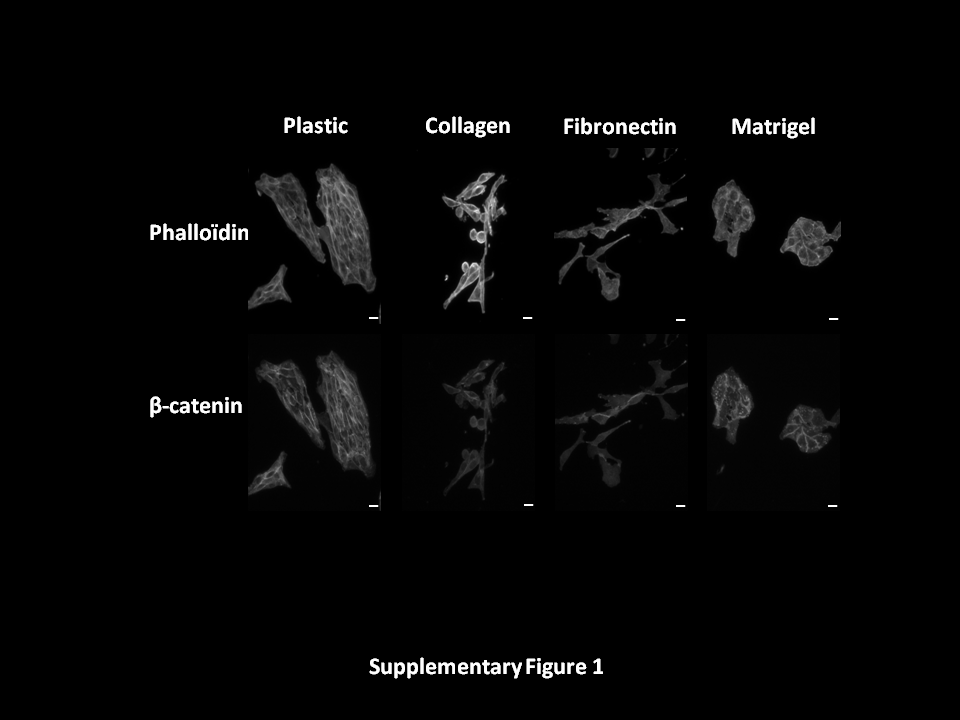

Supplement: Supplementary Figure 1 — Effect of the different ECM components on the phenotype of Renca cells. Cells were cultured for 24 h on uncoated (Plastic) or Col 1, FN1, or Matrigel glass coverslips, fixed and stained with β-catenin antibody or with phalloïdin (filamentous actin). Bar: 20 μm. N = 3. [file Image_1.TIF]

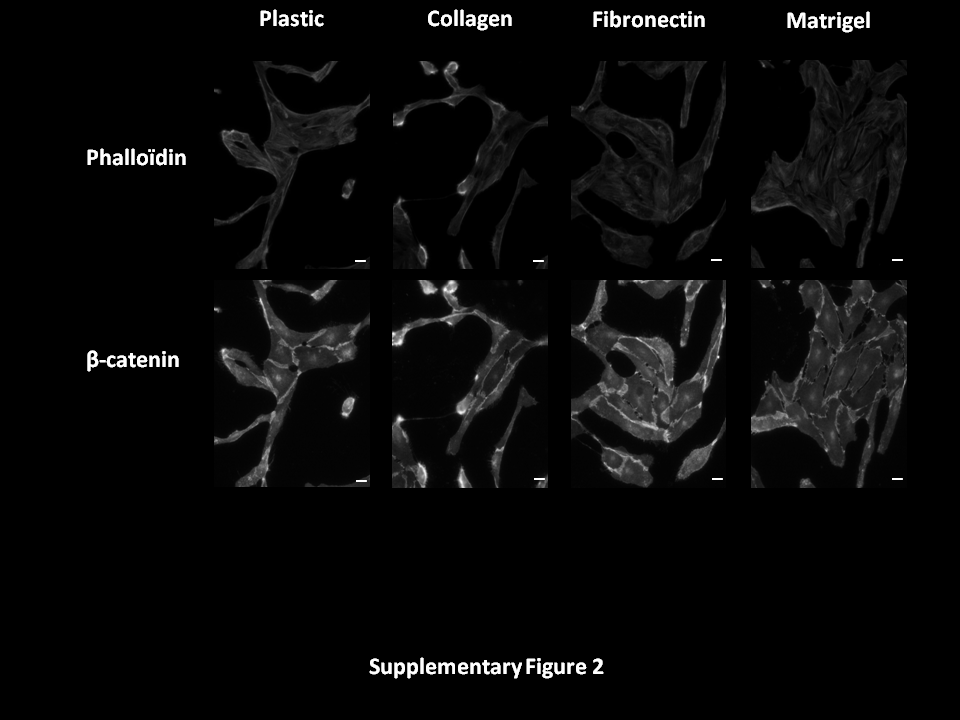

Supplement: Supplementary Figure 2 — Effect of the different ECM components on the phenotype of 786-O cells. Cells were cultured for 24 h on uncoated (Plastic) or Col 1, FN1, or Matrigel glass coverslips, fixed and stained with β-catenin antibody or with phalloïdin (filamentous actin). Bar: 20 μm. N = 3. [file Image_2.TIF]

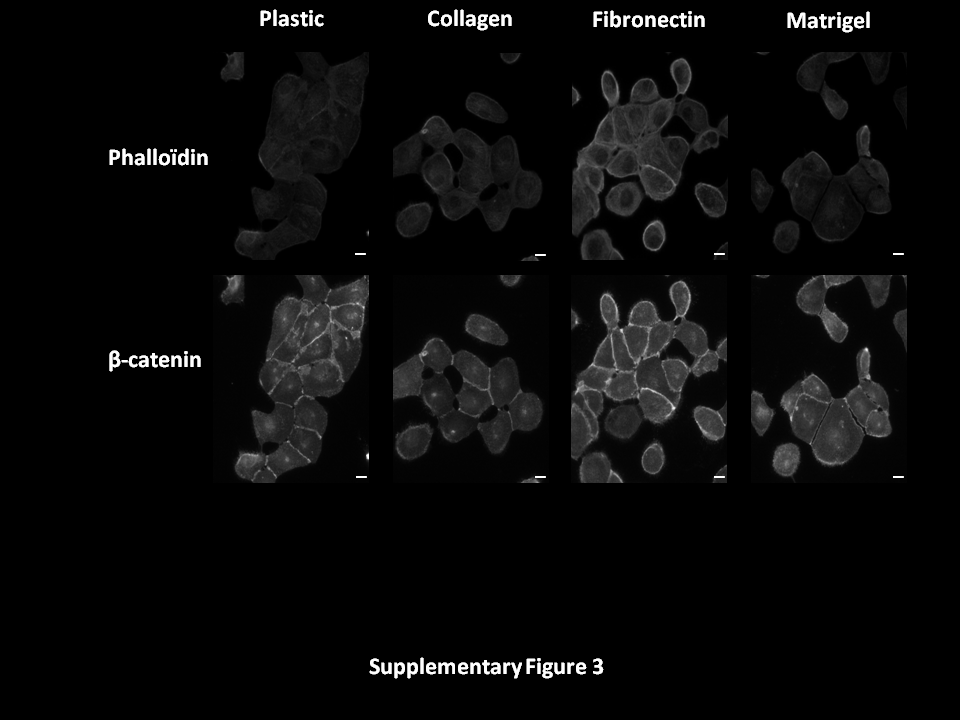

Supplement: Supplementary Figure 3 — Effect of the different ECM components on the phenotype of Caki-2 cells. Cells were cultured for 24 h on uncoated (Plastic) or Col 1, FN1, or Matrigel glass coverslips, fixed and stained with β-catenin antibody or with phalloïdin (filamentous actin). Bar: 20 μm. N = 3. [file Image_3.TIF]

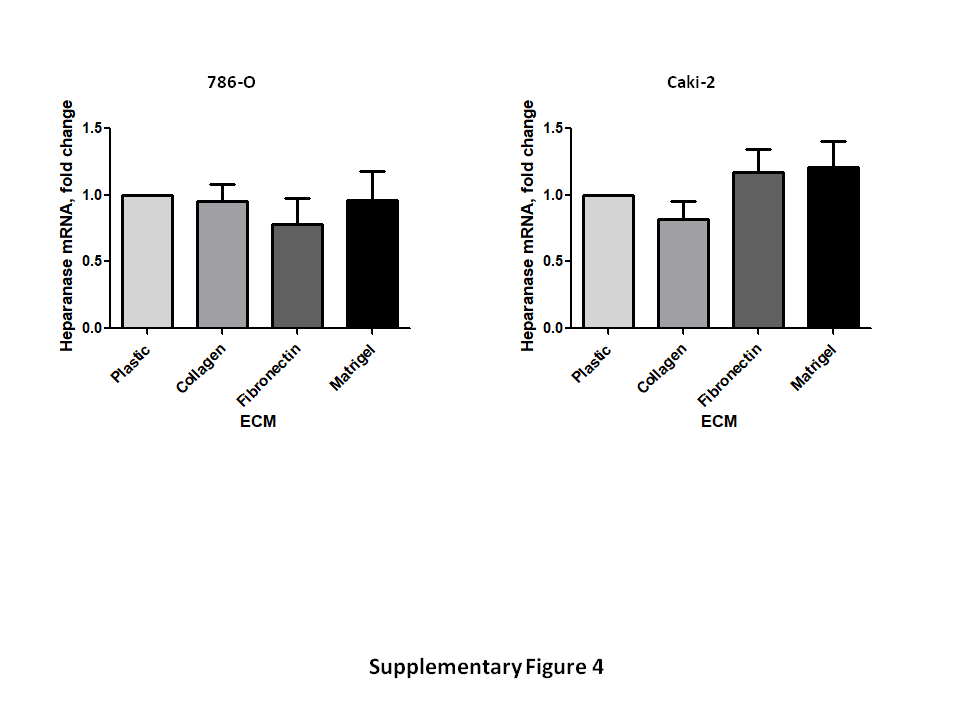

Supplement: Supplementary Figure 4 — Effect of the different ECM components on heparanase mRNA expression. Relative mRNA levels for heparanase were assessed by RT-qPCR after 24 h of RCC cells cultured on plastic, Col 1, FN1, or Matrigel. No heparanase mRNA were detected in Renca cells. N = 4. [file Image_4.TIF]

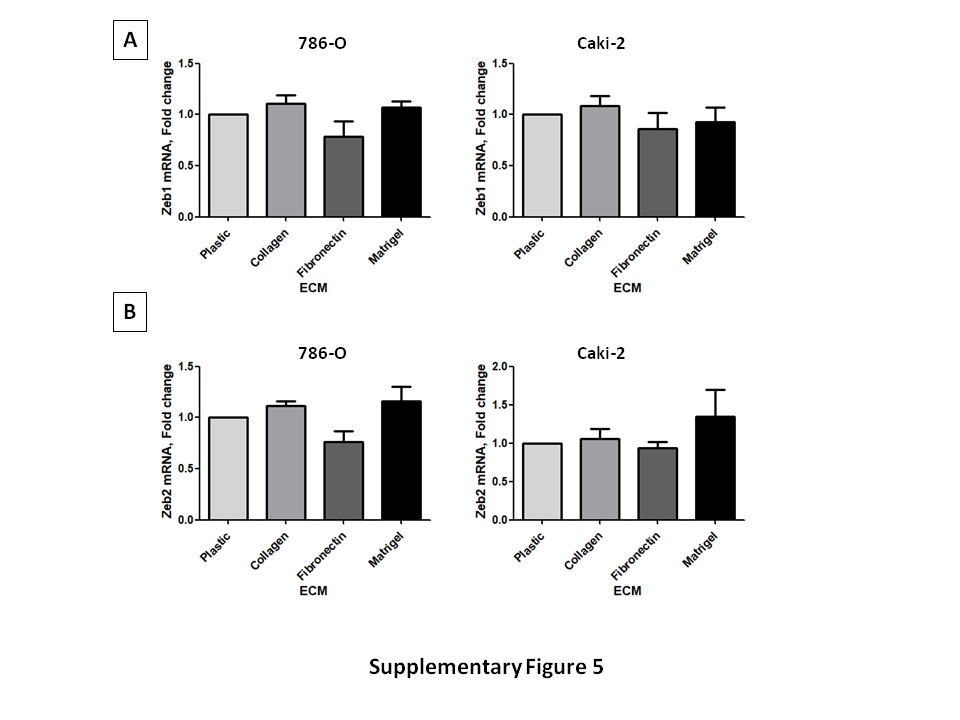

Supplement: Supplementary Figure 5 — Effect of the different ECM components on the expression of Zeb1 and 2, two transcription factors implicated in EMT. Relative mRNA levels for Zeb1 (A) and Zeb2 (B) were assessed by RT-qPCR after 24 h of RCC cells cultured on plastic, Col 1, FN1, or Matrigel. 786-O cells: N = 4. Caki-2 cells: N = 5. [file Image_5.TIF]

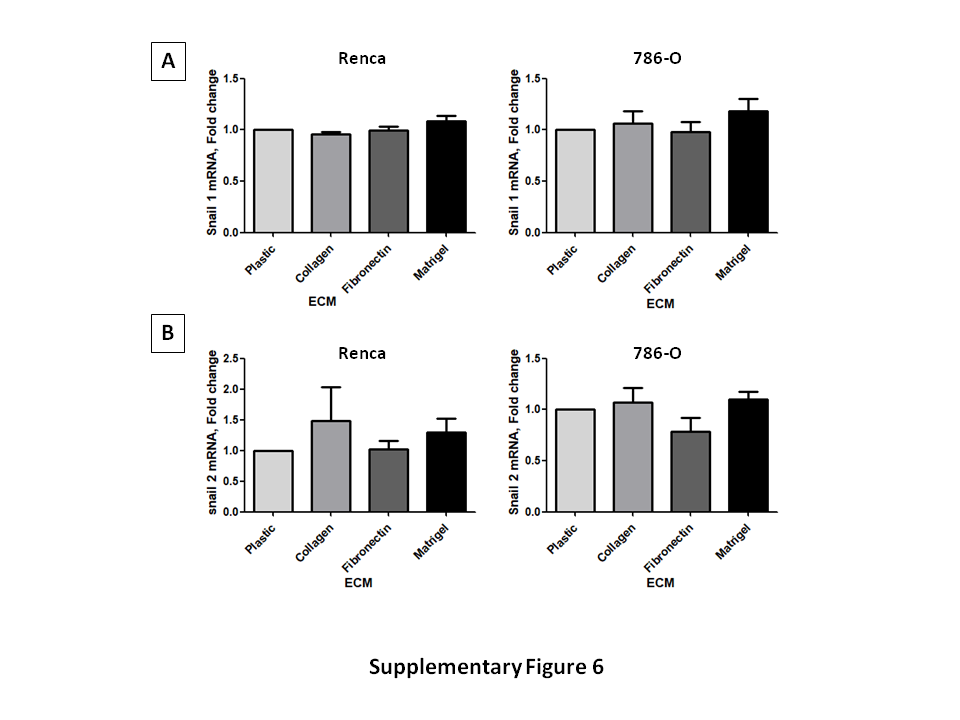

Supplement: Supplementary Figure 6 — Effect of the different ECM components on the expression of Snail 1 and 2, two transcription factors implicated in EMT. Relative mRNA levels for Snail 1 (A) and Snail 2 (B) were assessed by RT-qPCR after 24 h of RCC cells cultured on plastic, Col 1, FN1 or Matrigel. Renca cells, N = 7. 786-O cells: N = 4. [file Image_6.TIF]
